# Comparison of integron mediated antimicrobial resistance in clinical isolates of *Escherichia coli* from urinary and bacteremic sources

**DOI:** 10.1186/s12866-024-03250-3

**Published:** 2024-03-28

**Authors:** Gauri Kumar, Keshava Balakrishna, Chiranjay Mukhopadhyay, Vandana Kalwaje Eshwara

**Affiliations:** 1 Department of Microbiology, Kasturba Medical College, Manipal, Manipal Academy of Higher Education, Manipal, 576104 Karnataka India; 2 Department of Civil Engineering, Manipal Institute of Technology, Manipal Academy of Higher Education, Manipal, 576104 Karnataka India; 3 Center for Antimicrobial Resistance and Education (CARE), Manipal Academy of Higher Education, Manipal, 576104 Karnataka India; 4 Center for Emerging and Tropical Diseases (CETD), Manipal Academy of Higher Education, Manipal, 576104 Karnataka India

**Keywords:** Antimicrobial resistance, *Escherichia coli*, Class 1 integrons, Urinary *E. coli*, Bacteremic *E. coli*

## Abstract

**Background:**

Antimicrobial resistance (AMR) is a global threat driven mainly by horizontal gene transfer (HGT) mechanisms through mobile genetic elements (MGEs) including integrons. The variable region (VR) of an integron can acquire or excise gene cassettes (GCs) that confer resistance to antibiotics based on the selection pressure. *Escherichia coli* plays a significant role in the genetic transfer of resistance determinants to other Gram-negative bacteria. Current study is aimed to detect and compare integron-mediated resistance in clinical isolates of *E. coli*. Unique isolates of *E. coli* from urine or blood cultures were studied for their antimicrobial resistance patterns and integrons were detected using polymerase chain reaction assays followed by Sanger sequencing of GCs.

**Results:**

During the study period, a total of 470 *E. coli* isolates were obtained, 361 (76.8%) from urinary and 109 (23.1%) from bacteremic sources. Class 1 integrons were detected in 66 (18.2%) and 26 (23.8%) isolates respectively. Urinary isolates of *E. coli* harbouring Class 1 integrons demonstrated significantly higher rates of resistance (*p* < 0.05) for most antibiotics (12/16, 75%) compared to integron negative isolates. Although not statistically significant, similar differences were observed in bacteremic isolates. Among the urinary isolates, 27 (40.9%) had a VR, in which the most common GC array detected was *DfrA17-AadA5* (*n* = 14), followed by *DfrA5* (*n* = 4) and *DfrA12* (*n* = 3). Among bacteremic isolates, only 4 (15.3%) had a VR, all of which were carrying *DfrA17*. The detected GC array correlated with the respective isolates’ phenotypic resistance patterns.

**Conclusion:**

We found a strong correlation between integron positivity and trimethoprim resistance among *E. coli* from urinary sources. Although higher rates of resistance were observed in bacteremic isolates, they mostly carried empty integrons.

## Background

The imminent global threat of antimicrobial resistance (AMR) is particularly important due to the acquisition and transfer of resistance genes among bacteria harbored in humans, animals, and the environment. Among several human pathogenic bacteria, *Escherichia coli*, an important member of Enterobacterales, plays a major role in the genetic transfer of resistance to other Gram-negative bacteria [1, 2]. The spread of resistance mainly occurs through horizontal gene transfer (HGT) which is mediated by certain mobile genetic elements (MGEs) that promote intercellular and intracellular mobility of DNA such as insertion sequences (IS), transposons, plasmids, integrons, etc. While IS and transposons are mobile and can be inserted into random locations along with the resistance genes they carry, elements such as integrons depend on site-specific recombination to mobilise their resistance genes. Hence they can become mobile when associated with other MGEs such as transposons [3].

Integrons are considered a major driver contributing to the evolution and spread of resistance as they can acquire various unrelated antimicrobial resistance genes (ARGs) enabling multidrug resistance in bacteria. They contain a recombination system that is site-specific allowing them to capture, rearrange, and exchange gene cassettes (GCs). All integrons possess three elements for their proper functioning- tyrosine recombinase integrase encoded by the *intI* gene, a recombination site *attI*, and a constitutive promoter Pc found upstream of the site of integration and is required for expression of the GCs [4]. The GCs embedded within the integron are the variable sequences as they can be excised or acquired, depending on the requirement [3, 5].

Among the 5 classes, Class 1 integron is the most ubiquitous found in diverse bacterial genera such as *E. coli*, *Salmonella, Shigella, Vibrio, Campylobacter*, and *Pseudomonas*. Class 1 integrons are associated with transposon Tn402, usually inserted within a larger transposon Tn21. Integron-positive clinical bacterial strains usually carry less than 5 cassettes in varied combinations [6].

We carried out this study to understand the occurrence of class 1 integrons in clinical isolates of *E. coli* obtained from urine cultures or blood cultures (henceforth referred to as urinary and bacteremic isolates respectively) and to identify the GCs carried in their variable region (VR). The findings were further correlated with the phenotypic resistance patterns of the isolates.

## Methods

The study was conducted in the Microbiology laboratory attached to a University teaching hospital in the southwest region of India. Unique clinical isolates of *E. coli* obtained from urine cultures or blood cultures were collected between March 2022 and February 2023. Isolates were identified by MALDI-TOF (Vitek-MS) and antimicrobial susceptibility tests (AST) were performed using the VITEK 2 system. Fresh isolates were subcultured once to check purity, and DNA was extracted immediately by heat lysis method and stored at -20 °C for molecular assays [7].

All isolates were screened for the presence of class 1 integrons. A single-plex polymerase chain reaction (PCR) assay was designed using the primers *IntI-1* as previously described [7]. Twenty-five µL of the reaction constituted 2x PCR MasterMix (ThermoFisher), 25 pmol of forward and reverse primers and 3 µL (600 pg) of template DNA. PCR amplification was carried out using the following conditions: initial denaturation at 94 °C for 5 min followed by 35 cycles of 30 s of denaturation at 94 °C, 30 s of annealing at 54 °C, and 60 s of extension at 72 °C, with a final extension at 72 °C for 7 minutes [7]. An isolate confirmed to contain the integrase gene by sequencing was used as positive control.

Further, amplification of the VR of these integrons was performed to determine the gene cassettes they were carrying. Isolates positive for class 1 integron were subjected to PCR using primers previously described [7]. The reaction mixture was prepared similarly. The PCR parameters were also similar to that mentioned above, with the exception of annealing temperature set at 53 °C.

The PCR products were purified and sequenced using Sangers technique. The sequences obtained were analyzed by comparing using BLAST software (National Center for Biotechnology Information) [8].

### Statistical analysis

Antimicrobial resistance rates of the isolates were calculated as frequencies and percentages. Comparison of resistance rates between urinary and bacteremic *E. coli* isolates and also between integron positive and negative isolates was performed using Chi-square test or Fishers exact test, as appropriate, and was considered significant at *p* < 0.05. All analysis was done using RStudio v 4.3.0.

## Results

A total of 470 *E. coli* isolates were obtained, 361 (76.8%) from urinary sources and 109 (23.1%) from bacteremic sources. Bacteremic isolates demonstrated higher resistance rates to most of the antimicrobial agents tested compared to urinary isolates although a statistically significant difference was noted for antimicrobial agents belonging to cephalosporin class, nalidixic acid, trimethoprim/sulphamethoxazole, and ciprofloxacin.

Class 1 integrons were detected in 66/361 (18.2%) urinary isolates and 26/109 (23.8%) bacteremic isolates. Among the urinary *E.coli*, isolates harbouring class 1 integrons, demonstrated higher resistance rates to most (12/16, 75%) antimicrobial agents tested as opposed to the isolates not harbouring integrons. Although similar findings were observed in bacteremic isolates, the difference was not statistically significant. The detailed results are presented in Table 1.

VRs of the integrons were characterized using Sangers sequencing technique. Of the 66 integron positive urinary *E. coli*, the VR was successfully amplified and sequenced in 27 (40.9%) isolates. The most common GC array detected was *DfrA17-AadA5* (*n* = 14/ 27), followed by *DfrA5* (*n* = 4/27) and *DfrA12* (*n* = 3/27). Further details are presented in Table 2. All 25 isolates carrying *Dfr* GCs were resistant to trimethoprim sulphamethoxazole.

Only 4 (15.4%) of the 26 integron-positive isolates from bacteremic sources had VR. The GC identified was *DfrA17*. All 4 isolates also were resistant to trimethoprim sulphamethoxazole.

A positive correlation between the presence of integrons, presence of the *Dfr* genes in their VR, and resistance rates against trimethoprim sulphamethoxazole was noted in isolates from both clinical sources.

**Table 1:** Comparison of resistance rates of *E. coli* isolated from urine and blood cultures; and between integron positive and negative isolates from both these sources

Antibiotics	Overall resistance of *E. coli* from all isolates *n* = 470	Resistance rates of *E. coli* from urine cultures *n* = 361 (%)	Resistance rates of *E. coli* from blood cultures *n* = 109 (%)	***p*** value	Resistance rate of *E. coli *from urine cultures			Resistance rates of *E. coli* from blood cultures		
					Integron positive *n* = 66 (%)	Integron negative *n* = 295 (%)	***p*** value	Integron positive *n* = 26 (%)	Integron negative *n* = 83 (%)	***p*** value
Ampicillin	210 (77.4)#	161 (73.9)$	49 (92.5)^	0.948	23 (82.1)°	138 (72.6)¥	0.285	12 (92.3)Ω	37 (92.5)□	1
Amikacin	28 (6)	19 (5.3)	9 (8.3)	0.247	8 (12.1)	11 (3.7)	0.006*	1 (3.8)	8 (9.6)	0.683
Amoxicillin clavulanic acid	211 (44.9)	159 (44)	52 (47.7)	0.501	38 (57.6)	121 (41)	0.014*	15 (57.7)	37 (44.6)	0.243
Cefepime	182 (38.7)	123 (34.1)	59 (54.1)	< 0.001*	33 (50)	90 (30.5)	0.003*	17 (65.4)	42 (50.6)	0.187
Cefoperazone sulbactam	71 (15.1)	52 (14.4)	19 (17.4)	0.439	19 (28.8)	33 (11.2)	< 0.01*	6 (23.1)	13 (15.7)	0.385
Ceftriaxone	324 (68.9)	236 (65.4)	88 (80.7)	0.002*	47 (71.2)	189 (64.1)	0.27	21 (80.8)	67 (80.7)	1
Cefuroxime	353 (75.1)	258 (71.5)	95 (87.2)	0.001*	52 (78.8)	206 (69.8)	0.175	23 (88.5)	72 (86.7)	1
Ciprofloxacin	359 (76.4)	262 (72.6)	97 (89)	< 0.01*	61 (92.4)	201 (68.1)	< 0.01*	24 (92.3)	73 (88)	0.727
Ertapenem	59 (12.6)	43 (11.9)	16 (14.7)	0.445	15 (22.7)	28 (9.5)	0.003*	5 (19.2)	11 (13.3)	0.452
Gentamicin	128 (27.2)	98 (27.1)	30 (27.5)	0.938	21 (31.8)	77 (26.1)	0.345	6 (23.1)	24 (28.9)	0.561
Imipenem	47 (10)	35 (9.7)	12 (11)	0.689	13 (19.7)	22 (7.5)	0.002*	3 (11.5)	9 (10.8)	1
Meropenem	46 (9.8)	36 (10)	10 (9.2)	0.806	13 (19.7)	23 (7.8)	0.004*	2 (7.7)	8 (9.6)	1
Nalidixic acid	226 (83.3)#	178 (81.7)$	48 (90.6)^	< 0.001*	27 (96.4)°	151 (79.5)¥	0.034*	12 (92.3) Ω	36 (90)□	1
Nitrofurantoin	29 (10.7)#	17 (7.8)$	12 (22.6)^	0.002*	6 (21.4)°	11 (5.8)¥	0.012*	5 (38.5) Ω	7 (17.5)□	0.117
Piperacillin tazobactam	105 (22.3)	77 (21.3)	28 (25.7)	0.345	26 (39.4)	51 (17.3)	< 0.01*	10 (38.5)	18 (21.7)	0.088
Trimethoprim sulphamethoxazole	219 (46.6)	157 (43.5)	62 (56.9)	0.014*	55 (83.3)	102 (34.6)	< 0.01*	19 (73.1)	43 (51.8)	0.056

^#^Overall, only 271 out of 470 isolates were tested against these antibiotics; ^$^Only 218 out of 361 isolates were tested against these antibiotics; ^^^Only 53 out of 109 isolates were tested against these antibiotics; °Only 28 out of 66 isolates were tested against these antibiotics; ^¥^Only 190 out of 295 isolates were tested against these antibiotics; ^Ω^Only 13 out of 26 isolates were tested against these antibiotics; ^□^Only 40 out of 83 isolates were tested against these, *Significant *p* value < 0.05

**Table 2:** Gene cassettes detected in integron positive isolates obtained from urine and blood cultures

Source	Number of integron positive isolates (%)	Number of isolates with VR (%)	GC^a^	Number of isolates with GC (%)
***E. coli*isolated from urine cultures** (*n*** = 361)**	66 (18.2)	27 (40.9)	*DfrA17-AadA5*	14 (51.8)
			*DfrA5*	4 (14.8)
			*DfrA12*	3 (11.1)
			*DfrA7*	2 (7.4)
			*DfrA17*	2 (7.4)
			*AadA2*	1 (3.7)
			*AadA5*	1 (3.7)
***E. coli*isolated from blood cultures** (*n*** = 109)**	26 (23.8)	4 (15.4)	*DfrA17*	4 (100)

^a^*DrfA17, DfrA5, DfrA12, DfrA7* encode dihydrofolate reductase conferring resistance to trimethoprim; *AadA2, AadA5* encode aminoglycoside adenyltransferase conferring resistance to spectinomycin/streptomycin [9, 10]

## Discussion

Our results present an important genetic mechanism of antimicrobial resistance, via integrons which can harbour and disseminate resistance genes in the commonest human pathogen *E. coli*. We found an overall higher antimicrobial resistance rates in bacteremic *E. coli* compared to urinary *E. coli*. While class 1 integrons were detected in a higher proportion of bacteremic *E. coli*, the VR and the GC were relatively more frequent in urinary *E. coli*. Of all the antimicrobial agents tested, the highest rates of resistance were observed for the drugs belonging to fluoroquinolones, cephalosporins, beta-lactam beta-lactamase inhibitors, trimethoprim sulphamethoxazole, and aminoglycosides.

Sufficient molecular findings have shown that AMR is commonly achieved by the acquisition of pre-existing resistance determinants that are amplified as a result of selection pressure. This acquisition is largely due to MGEs, such as integrons, which can promote intracellular and intercellular DNA mobility [11, 12]. The structure of the integron is such that it has a VR flanked by two conserved sequences (CS). The 5’CS includes the *intI* gene, *attI* gene, and Pc (required for recombination and expression of GCs), whereas the 3’CS consists of *qacEΔ1A* gene (conferring resistance to certain detergents), *sulI* gene (conferring resistance to sulphonamides) and an *orf* gene (unknown function). The VR in the middle is where the GCs are located [4].

A comprehensive meta-analysis conducted by Halaji et al. (2020) revealed the discrepancy in reports of the prevalence of integrons in *E. coli* from patients with UTIs. In their review, they noted the prevalence of integrons in UPEC isolates to be between 15% and 90%. This wide range could be due to the origin of isolates and geographical distribution. While it would be difficult to observe a pattern due to differences in methodologies of studies and underreporting, it has been noticed that Asian countries report an average of 54% of UPEC isolates to carry class 1 integrons [13]. In our study, cumulatively, approximately 20% of the isolates were positive for class 1 integrons.

The importance and danger of integrons lie in understanding the reason behind their mobilisation. Most integrase genes carry LexA binding sites close to the promoter region which can be regulated by the LexA protein of the host. This protein is a repressor of the SOS response (physiological stress response) suggesting that induction of SOS could increase transcription of the integrase gene and hence increase activity of the integrase which involves GC rearrangements. It is due to this that the integron system can adapt to environmental changes. The SOS response system controls processes that promote an exchange of GCs and the mobilisation of integrons [4]. In clinical settings, the SOS response is triggered by the use of antibiotics, especially trimethoprim, fluoroquinolones, and beta-lactams which induce integrase expression. The most common GCs identified in our study belong to some of these classes, such as trimethoprim, which emphasises the fact that some antibiotics could directly promote the acquisition and dissemination of resistance determinants.

GCs are small mobile elements (∼0.5-1 kb) that are non-replicative and are most often found inserted in an integron. Their structure is such that they do not have a promoter, but consist of an open reading frame (ORF) and a cassette recombination site (*attC*). Production of the site-specific tyrosine recombinase produced by the *intI* gene in the structure of the integron facilitates recombination between *attI* site of the integron and *attC* site of the GC. Promoterless, the GC depends on the Pc of the integron for its expression after acquisition. Multiple GCs can accumulate in an integron (known as an array), however, GCs away from the Pc may not be expressed [3, 4, 12]. High rates of resistance against trimethoprim sulphamethoxazole were observed in our study isolates which could be explained by the inherent presence of the *sul1* gene in the integron [3].

The GCs detected in this study belong to gene families that encode for resistance to trimethoprim (*Dfr*) and aminoglycosides such as streptomycin and spectinomycin (*Aad*) [9, 10]. The most common GC array in our study was also found to be *DfrA17*-*Aad* (51.8%) which can have negative impacts on the therapeutic use of antibiotics. Additionally, detection of the *aadA* gene present upstream of the *dfrA* gene corroborates the results of previous studies suggesting that the *AadA* gene is the first cassette acquired and/or maybe more stable than other GCs [10]. The individual GCs belonging to the *Dfr* and *AadA* families detected in our study are also amongst the most commonly observed genes to be found associated with class 1 integrons in clinical isolates which could also indicate the historical selection pressure that was exerted when these drugs were employed more widely in the past [3, 9, 14, 15].

Class 1 integrons have been seen to carry a wide selection of GCs conferring resistance to different classes of drugs [13, 16]. Our study isolates revealed significantly higher rates of resistance (*p* < 0.05) for most antimicrobial drugs, except ampicillin, ceftriaxone, cefuroxime and gentamicin, in integron-positive compared to integron-negative isolates from urinary sources. Although the GCs conferring resistance to these classes of drugs were not found in the VR of the integrons, it is possible that they were localised outside the integron [3, 9, 14, 16].

A potential reason for the failure of amplification of the VR in some strains could be the absence of the 3’ conserved sequence characterised by the *sul1* gene. This could be the case in some isolates that are integron positive but are susceptible to sulphamethoxazole. However, for the majority of isolates, a further genetic exploration is required to explain this. It is also possible that the GCs were excised from the integron structure due to the lack of selection pressure exerted by antibiotic pressure [9, 16, 17].

A limitation of our study is that presence of GCs in the integron structure can only support the resistance patterns of trimethoprim sulphamethoxazole seen in urinary and bacteremic isolates, but cannot explain the entirety of the resistance pattern observed in them. Further detailed genotypic investigations are required to detect presence of these genes elsewhere in the genome of the bacteria. However, through this study, we have understood the prevalence of class 1 integrons in the most common organism associated with urinary tract infections and bacteremia and the potential mechanisms of AMR development and spread.

## Conclusion

Integron associated resistance is common against trimethoprim in urinary *E. coli.* Despite showing higher antimicrobial resistance rates, the bacteremic isolates mostly harboured empty integrons. Regular monitoring of the mechanisms of HGT are essential to develop the AMR control strategies.

## Data Availability

All data and analysis in this study has been mentioned in the manuscript.

## Abbreviations

AMR: Antimicrobial Resistance
ARG: Antimicrobial Resistance Genes
AST: Antimicrobial Susceptibility Testing
CS: Conserved Sequence
GCs: Gene Cassettes
HGT: Horizontal Gene Transfer
MALDI-TOF: Matrix-Assisted Laser Desorption Ionization–Time-Of-Flight
MGE: Mobile Genetic Elements
PCR: Polymerase Chain Reaction
UPEC: Uropathogenic *E. coli*
VR: Variable Region
